# Cardiac stasis imaging, stroke, and silent brain infarcts in patients with nonischemic dilated cardiomyopathy

**DOI:** 10.1152/ajpheart.00245.2024

**Published:** 2024-06-07

**Authors:** Elena Rodríguez-González, Pablo Martínez-Legazpi, Ana González-Mansilla, M. Ángeles Espinosa, Teresa Mombiela, Juan A. Guzmán De-Villoria, Maria Guadalupe Borja, Fernando Díaz-Otero, Rubén Gómez de Antonio, Pilar Fernández-García, Ana I. Fernández-Ávila, Cristina Pascual-Izquierdo, Juan C. del Álamo, Javier Bermejo

**Affiliations:** ^1^Department of Cardiology, Hospital General Universitario Gregorio Marañón, Madrid, Spain; ^2^Department of Medicine, Facultad de Medicina, Universidad Complutense, Madrid, Spain; ^3^Instituto de Investigación Sanitaria Gregorio Marañón, Madrid, Spain; ^4^Centro de Investigación Biomédica en Red de Enfermedades Cardiovasculares, Madrid, Spain; ^5^Department of Mathematical Physics and Fluids, Facultad de Ciencias, Universidad Nacional de Educación a Distancia, Madrid, Spain; ^6^Department of Radiology, Hospital General Universitario Gregorio Marañón, Madrid, Spain; ^7^Centro de Investigación Biomédica en Red de Salud Mental, Madrid, Spain; ^8^Department of Mechanical and Aerospace Engineering, University of California San Diego, La Jolla, California, United States; ^9^Department of Neurology, Hospital General Universitario Gregorio Marañón, Madrid, Spain; ^10^Department of Hematology, Hospital General Universitario Gregorio Marañón, Madrid, Spain; ^11^Division of Cardiology, Department of Mechanical Engineering, Center for Cardiovascular Biology, University of Washington, Seattle, Washington, United States

**Keywords:** blood stasis, cardioembolism, echocardiography, heart failure, ischemic stroke

## Abstract

Cardioembolic stroke is one of the most devastating complications of nonischemic dilated cardiomyopathy (NIDCM). However, in clinical trials of primary prevention, the benefits of anticoagulation are hampered by the risk of bleeding. Indices of cardiac blood stasis may account for the risk of stroke and be useful to individualize primary prevention treatments. We performed a cross-sectional study in patients with NIDCM and no history of atrial fibrillation (AF) from two sources: *1*) a prospective enrollment of unselected patients with left ventricular (LV) ejection fraction <45% and *2*) a retrospective identification of patients with a history of previous cardioembolic neurological event. The primary end point integrated a history of ischemic stroke or the presence intraventricular thrombus, or a silent brain infarction (SBI) by imaging. From echocardiography, we calculated blood flow inside the LV, its residence time (*T*_R_) maps, and its derived stasis indices. Of the 89 recruited patients, 18 showed a positive end point, 9 had a history of stroke or transient ischemic attack (TIA) and 9 were diagnosed with SBIs in the brain imaging. Averaged *T*_R_, $\bar{T_{\text{R}}},\;$ performed well to identify the primary end point [AUC (95% CI) = 0.75 (0.61–0.89), *P* = 0.001]. When accounting only for identifying a history of stroke or TIA, AUC for $\bar{T_{\text{R}}}$ was 0.92 (0.85–1.00) with odds ratio = 7.2 (2.3–22.3) per cycle, *P* < 0.001. These results suggest that in patients with NIDCM in sinus rhythm, stasis imaging derived from echocardiography may account for the burden of stroke.

**NEW & NOTEWORTHY** Patients with nonischemic dilated cardiomyopathy (NIDCM) are at higher risk of stroke than their age-matched population. However, the risk of bleeding neutralizes the benefit of preventive oral anticoagulation. In this work, we show that in patients in sinus rhythm, the burden of stroke is related to intraventricular stasis metrics derived from echocardiography. Therefore, stasis metrics may be useful to personalize primary prevention anticoagulation in these patients.

## INTRODUCTION

Cardioembolic stroke is associated with a high in-hospital mortality (1). Around 20% of these strokes occur in patients with sinus rhythm and structural heart disease. Left ventricular (LV) systolic dysfunction, due to either ischemic or nonischemic dilated cardiomyopathy (NIDCM), is the most frequent finding in these patients (2). In fact, patients with NIDCM show an annual incidence of stroke three to eight times higher than the age-matched population (3). Several studies have also demonstrated a high prevalence of silent brain infarction (SBI) in the setting of dilated cardiomyopathy, even in the absence of atrial fibrillation (AF) (4). SBIs are a documented source of disability and mortality, and both their presence and number are highly sensitive predictors of clinical stroke (5).

Randomized controlled clinical trials have failed to demonstrate a significant benefit of oral anticoagulation in non-AF patients with heart failure (HF) and reduced ejection fraction, as the reduction of thrombotic events was counterbalanced by the incidence of bleeding complications (6). Trials aimed at secondary prevention after embolic strokes of unknown source have also been neutral (7). Therefore, there is an unmet medical need for developing robust markers to address the risk of cardioembolism in non-AF patients with HF.

In NIDCM, global and regional LV chamber abnormalities induce an abnormal blood flow transit inside the chamber that leads to regions of increased blood stasis (8). Combined with the prothrombotic state caused by HF neurohormonal activation, blood stasis is a risk factor for cardioembolic events, even in the absence of AF (9). In recent years, computational image methods to quantify blood stasis inside the heart using conventional modalities have been developed (10, 11). The number of cycles spent by blood particles inside the LV (i.e., the residence time, *T*_R_) has been proposed as an image-based biomarker of stasis. Preliminary studies have shown the potential of the average *T*_R_ in the LV for addressing the risk of cardioembolism in patients with ventricular dysfunction after ST-elevation myocardial infarction (STEMI) (12, 13). This index has also been suggested to be a biomarker of the significance of coronary artery stenosis, atherosclerosis (14), and ventricular dysfunction (15). We hypothesized that elective stasis imaging may allow identifying patients with NIDCM at risk of cardioembolism, and by guiding prevention strategies, eventually improve outcomes. On these bases, we designed the ISBIDCM clinical trial, “Imaging Silent Brain Infarct in Dilated Cardiomyopathy” (NCT 03415789), to decipher the relationship of intraventricular stasis with the prevalence of SBIs, intraventricular mural thrombosis, and/or stroke in patients with NIDCM.

## METHODS

Inclusion criteria in the ISBIDCM trial were the diagnosis of NIDCM with an LV ejection fraction ≤45% (unrelated to ischemic cardiomyopathy), age ≥18 yr old, and no history of AF (either clinically or subclinically detected in Holter tests or by any cardiac monitoring device). Exclusion criteria were any contraindication for cardiac magnetic resonance (CMR) exam, any primary valve disease ≥3+ severity, a diagnosis of carotid artery disease with >50% stenosis, oral anticoagulation before enrollment, history of prothrombotic disease, and reluctance to sign the written informed consent.

The sample size for the ISBIDCM study was established based on an estimated prevalence of SBIs of 30–35% (4). However, after achieving the recruitment target, the number of patients with the primary end point was 11% (see *Study End Points*). As this markedly lowered the power of the study, the Steering Committee approved enriching the positive end-point population with an additional group of patients with NIDCM who had a medical history of ischemic neurological events and met the other inclusion criteria for the ISBIDCM study. Therefore, we added additional cases to the study by retrospectively screening patients from November 2019 to February 2022 with a history of stroke or transient ischemic attack. The Ethics Committee of the Hospital Gregorio Marañón approved the study and all patients provided written informed consent.

### Study End Points

**Figure 1.**
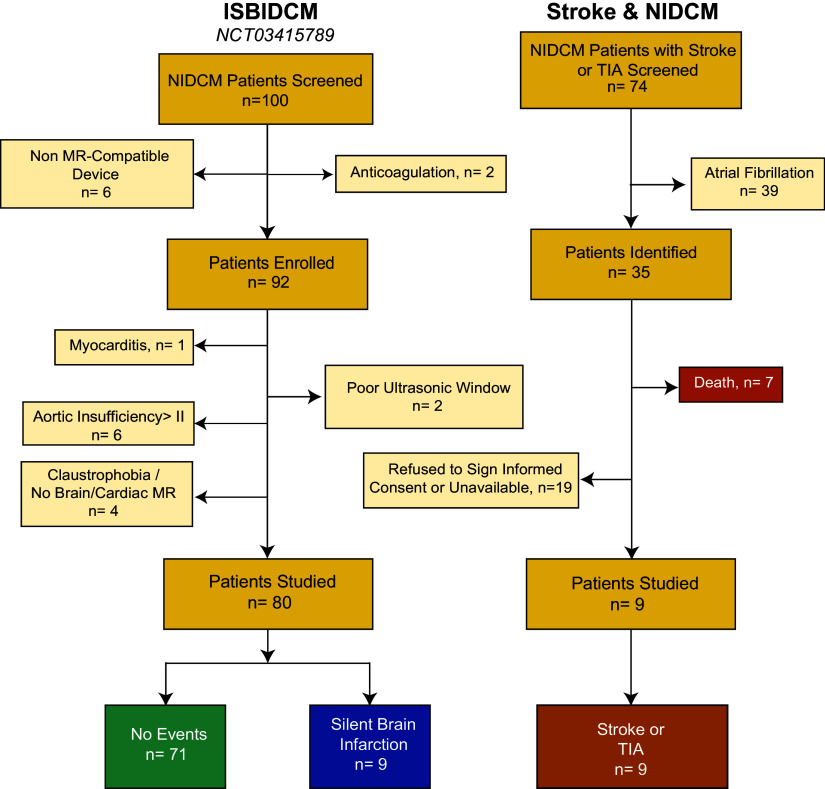
Flow diagram of patient populations and primary end points. TIA, transient ischemic attack; *n*, number of subjects.

The primary end point for the study was either *1*) the identification of an any-date SBI lesion in the brain MR examination, *2*) the presence of an intraventricular thrombus in any imaging test, or *3*) a clinical history of stroke or transient ischemic attack (TIA) (Fig. 1). Transcranial Doppler and carotid duplex ultrasonographic examinations, and 24-h Holter monitoring tests were performed in patients with the primary end point to rule out alternative causes of stroke, TIA, or SBI beyond cardioembolism.

### Ancillary Variables and Blood Biomarkers

In all patients, a comprehensive assessment of coagulation, fibrinolysis biomarkers, von Willebrand Factor, and ADAMTS13 activity was performed, and they underwent Beck Depression Inventory and the Mini-Mental State Examinations to screen for depression and dementia, respectively.

### Conventional Cardiac and Brain Imaging

All patients underwent an exhaustive echocardiographic exam. In patients with no history of ischemic neurological event, cardiac and brain magnetic resonance examinations were also performed.

### Cardiac Imaging

Echocardiographic examinations were performed at the enrollment using a Vivid 7 scanner and a phase-array 2–4-MHz transducer (GE Healthcare). We obtained three-dimensional (3-D) apical sequences to measure LV volumes and ejection fraction (EF). Longitudinal myocardial strain was measured from apical long-axis sequences using a six-segment model (EchoPac version 204, GE Healthcare). The E-wave propagation index, a reported biomarker of mural thrombosis, was computed as the E-wave velocity time integral over the LV long-axis length (16). The CMR imaging protocol included a cine steady-state free precession imaging of LV function (SENSE X 2, repetition time: 2.4 ms, echo time: 1.2 ms, average in-plane spatial resolution: 1.6 × 2 mm, 30 phases per cycle, 8-mm slice thickness without gap) and late enhancement imaging (3-D inversion-recovery turbo gradient echo sequence, prepulsed delay optimized for maximal myocardial signal suppression; 5-mm actual slice thickness, inversion time: 200–300 and 600 ms). Images were obtained in short axis (10–14 contiguous slices) and four-, two-, and three-chamber views. Late enhancement sequences were obtained 3 and 10 min after injection of 0.1 mmol/kg of gadobenate dimeglumine (ProHance, Bracco Imaging) to assess for mural thrombosis and quantify myocardial focal fibrosis.

### Brain Imaging

Brain MR was performed along CMR imaging and included sagittal T1-weighted images, axial diffusion weighted images, coronal T2-weigthed turbo spin echo (TSE), and axial FLAIR-T2-weighted images. Acute or subacute diffusion-weighted image strokes were adjudicated whenever focal lesions greater than 3 mm hyperintense on diffusion-weighted images with low apparent coefficient diffusion or pseudo normalization values were, respectively, identified. Chronic ischemic injury was diagnosed when the lesion was isointense to CSF on T2 and FLAIR weighted images appeared surrounded by an hyperintense lineal rim of gliosis and showed high apparent coefficient diffusion values. All studies were interpreted by expert neuroradiologists who also differentiated these types of lesions from dilated perivascular spaces based on their distribution and morphology.

### Stasis Mapping

From color-Doppler, we obtained two-dimensional, time-resolved (2-D + t) blood flow velocity vector fields in the apical long-axis view of the LV, using vector flow mapping (VFM), as described elsewhere (17). We used the VFM flow fields, to integrate forced advection equations to map and quantify the residence time, *T*_R_ (units of time), of the infinitesimal blood volumes inside the LV:

(*1*) $$\frac{\partial T_{\text{R}}}{\partial t}+\nabla \cdot \left(\vec{v}T_{\text{R}}\right)\;=\;1,\;$$

where $\vec{v\;}$ is the 2-D + *t* VFM velocity field at each point of space and time inside the LV. We integrated *Eq. 1* for spanning eight consecutive cardiac cycles, the time a normal LV takes to fully washout (18). At the end of the 8th beat, we collected the average $\bar{T_{\text{R}}}$ inside the LV. However, LV regional wall motion impairment may induce regions of stasis in its vicinity that may be overlooked by focusing only on $\bar{T_{\text{R}}}$. Therefore, we designated as stagnant regions those with $T_{\text{R}}$, >2 cardiac cycles and as severely stagnant regions those with *T*_R_ >6 cardiac cycles (12, 19). From those regions, we measured *1*) their size relative to total LV size and *2*) a stasis timescale, *T*_s_, that accounts for degree of distortion blood experience inside them. *T*_s_ was defined as the squared inverse of the second invariant of the symmetric strain tensor, $S_{i,j}\;=\;\left(\partial_{x,j}u_{i}+\partial_{x,i}u_{j}\right)/2$. Therefore, $T_{\text{s}}\;=\;1/\sqrt{{\text{Q}}_{\text{s}}},\;$ with ${\text{Q}}_{\text{s}}\;=\;trace\left(\;S_{i,j}^{2}\right)/2\;$(10, 20). We rather use *T*_s_ instead of the kinetic energy as it is plausible to encounter fluid regions with larger residence time moving with little distortion, similar to a rigid solid. Stagnant regions were automatically measured, and those not spanning a full cardiac cycle or <2% of LV area were discarded. Whenever more than one stagnant region was identified, only the larger was analyzed. The entire process was done blinded to the clinical events. We found no meaningful difference in the performance of calculating *T*_R_ in seconds instead of cardiac cycles to address the primary end point, because both *T*_R_ metrics correlated tightly (*R* = 0.94, pooled studies). The vector flow mapping flow fields, and the residence time maps, have been compared, with excellent agreement, with data from in silico experiments and from real patients using 4-D flow PC-MRI (17, 21–25). The interobserver and intraobserver reproducibility of *T*_R_ have been previously reported (intraclass correlation coefficient = 0.90 and 0.77, respectively) (18).

We designed a color-coded scale to generate video maps of *T*_R_ of blood in the LV. In each frame, the color scale represents the number of cardiac cycles a blood particle has spent inside the chamber; dark blue represents “fresh” blood recently entering the LV, whereas dark red represents stasis regions in which with blood is retained for at least four cardiac cycles. The evolution of blood residence time in the LV during the entire calculation period is shown in Supplemental Video S1 for a patient with a stroke and in Supplemental Video S2 for a patient without primary end point.

### Statistical Analysis

Data are described as median [interquartile range] except otherwise indicated. We used Wilcoxon rank-sum, χ^2^, and Fisher exact tests to compare quantitative variables and proportions, respectively. We used logistic regression to address the performance for identification of the primary end point, adjusting for clinical factors (see below). Odds ratios and their 95% confidence intervals (CIs) are reported for these models. To test the diagnostic performance, we used receiver-operating characteristic (ROC) analyses to compute the area under the curve (AUC), its 95% CI, and its statistical significance. Medians (and 95% CIs) of performance metrics were calculated by bootstrapping with 2,000 replicates. All statistical analyses were performed in *R* (v.4.1.3) and *P* values < 0.05 were considered significant.

## RESULTS

We included 89 patients (80 patients from the original ISBIDCM cohort and 9 patients with diagnosis of stroke or ischemic transient attack from the additional cohort), with a median age of 59 [49–69] yr old, and 37 (42%) were women. The primary end point was recorded in 18 patients (20%): 9 patients with a history of stroke or TIA, and 9 with identified SBIs (2 patients with 3 lesions, 1 patient with 2, and 6 patients with 1); no intraventricular thromboses were found. A total of 55% of the SBI were found in the cerebellum, three of them were located cortically, and one of the events had cortico-subcortical location. Carotid duplex and transcranial Doppler ruled out alternative etiologies of the neurological events in all patients with a primary end point.

There were no differences in age, sex, and other clinical variables as New York Heart Association (NYHA) class or current treatment among patients with and without the primary end point. However, patients with a positive primary end point showed more frequent diabetes mellitus and dyslipidemia (53 vs. 27 and 63 vs. 35%, respectively; all *P* < 0.05) and had higher CHA_2_DS_2_-VASc scores (3.5 [3–5] vs. 2.0 [2–3], *P* = 0.004) than those without an end point. Also, patients with stroke or SBI showed higher levels of creatinine (1.20 [0.88–1.42] vs. 0.87 [0.77–1.08] mg/dL, *P* = 0.01). Other hematological, coagulation and biochemical variables were similar in both groups (Table 1 and Supplemental Table S1). Patients with a primary end point showed lower Mini-Mental scores than patients without one, whereas there were no differences in the Beck Depression test.

**Table 1.** Clinical and imaging data

	Overall	End Point (−)	End Point (+)	*P* Value
*n*	89	71	18	
Age, yr	59 [49–69]	57 [48–65]	66 [55–77]	0.06
Females, *n* (%)	37 (42)	29 (41)	8 (44)	0.78
Clinical data				
Diabetes mellitus, *n* (%)	29 (32)	19 (27)	10 (53)	0.02*
Dyslipidemia, *n* (%)	37 (41)	25 (35)	12 (63)	0.01*
Hypertension, *n* (%)	48 (53)	34 (48)	14 (74)	0.06
CHA_2_DS_2_-VASc score	2 [2–4]	2 [2–3]	3.5 [3–5]	0.002*
Laboratory				
Hemoglobin, g/dL	14.1 [13.1–15.6]	14.1 [13.2–16.0]	14.0 [12.8–15.0]	0.52
Creatinine, mg/dL	0.91 [0.80–1.14]	0.87 [0.77–1.08]	1.20 [0.88–1.42]	0.01*
NT-proBNP, pg/mL	419 [199–1,013]	374 [197–878]	1,017 [385–1,657]	0.11
Blood biomarkers				
Prothrombin time, s	11.8 [11.3–12.5]	11.8 [11.3–12.5]	12.2 [10.8–12.3]	0.94
Von Willebrand activity, IU/dL	181 [107–205]	181 [107–202]	188 [177–229]	0.28
Activated partial thromboplastin time, s	29.8 [27.7–32.2]	29.9 [28.1–32.2]	29.3 [26.5–30.9]	0.34
ADAMS13 test, %	68 [58–83]	68 [56–83]	65 [65–84]	0.90
Echocardiography				
Heart rate, beats/min	71 [60–80]	70 [60–78]	81 [72–95]	0.10
LV end-diastolic volume index, mL/m^2^	69 [58–88]	69 [58–85]	71 [58–103]	0.39
LV end-systolic volume index, mL/m^2^	46 [33–61]	43 [33–59]	51 [32–68]	0.44
LV ejection fraction, %	37 [28–44]	37 [28–44]	36 [27–44]	0.76
E-wave velocity, cm/s	0.59 [0.48–0.73]	0.59 [0.48–0.73]	0.54 [0.50–0.70]	0.79
A-wave velocity, cm/s	0.71 [0.57–0.83]	0.72 [0.60–0.83]	0.62 [0.56–0.84]	0.38
Global peak systolic longitudinal strain, %	−10.1 [−10.3 to −9.7]	−10.1 [−10.3 to −9.7]	−10.1 [−10.3 to −9.9]	0.77
Apical peak systolic longitudinal strain, %	−10.8 [−13.8 to −10.1]	−10.8 [−13.8 to −10.1]	−10.8 [−13.8 to −10.8]	0.91
Stasis imaging				
Average blood residence time, cycles	1.95 [1.54–2.77]	1.85 [1.52–2.48]	2.79 [2.31–3.52]	0.001
Size of stagnant regions, %	37 [27–56]	36 [24–50]	56 [35–62]	0.009
Stasis timescale of stagnant regions, s	0.49 [0.37–0.69]	0.47 [0.36–0.63]	0.77 [0.49–1.57]	<0.001
Size of severely stagnant regions, %	4 [2–12]	4 [1–9]	13 [3–20]	0.007
Stasis timescale of severely stagnant regions, s	0.53 [0.34–0.88]	0.44 [0.32–0.66]	1.18 [0.63–2.28]	<0.001

Values are medians [interquartile range 25–75%] and *n* (%). LV, left ventricle.

* *P* < 0.05.

Traditional cardiac imaging indices showed no significant differences among patients with and without the primary end point. In fact, both groups showed nearly identical values of all systolic and diastolic variables (Table 1 and Supplemental Table S2). Left atrial diameter was similar in both groups (4.1 [3.6–4.5] vs. 4.0 [3.6–4.6] cm, *P* = 0.9), and no differences were found in focal fibrosis as detected by LGE in CMR. Also, the global and apical LV strains measured by echocardiography were similar in patients with and without the end point.

### Stasis Imaging and Primary End Point

**Figure 2.**
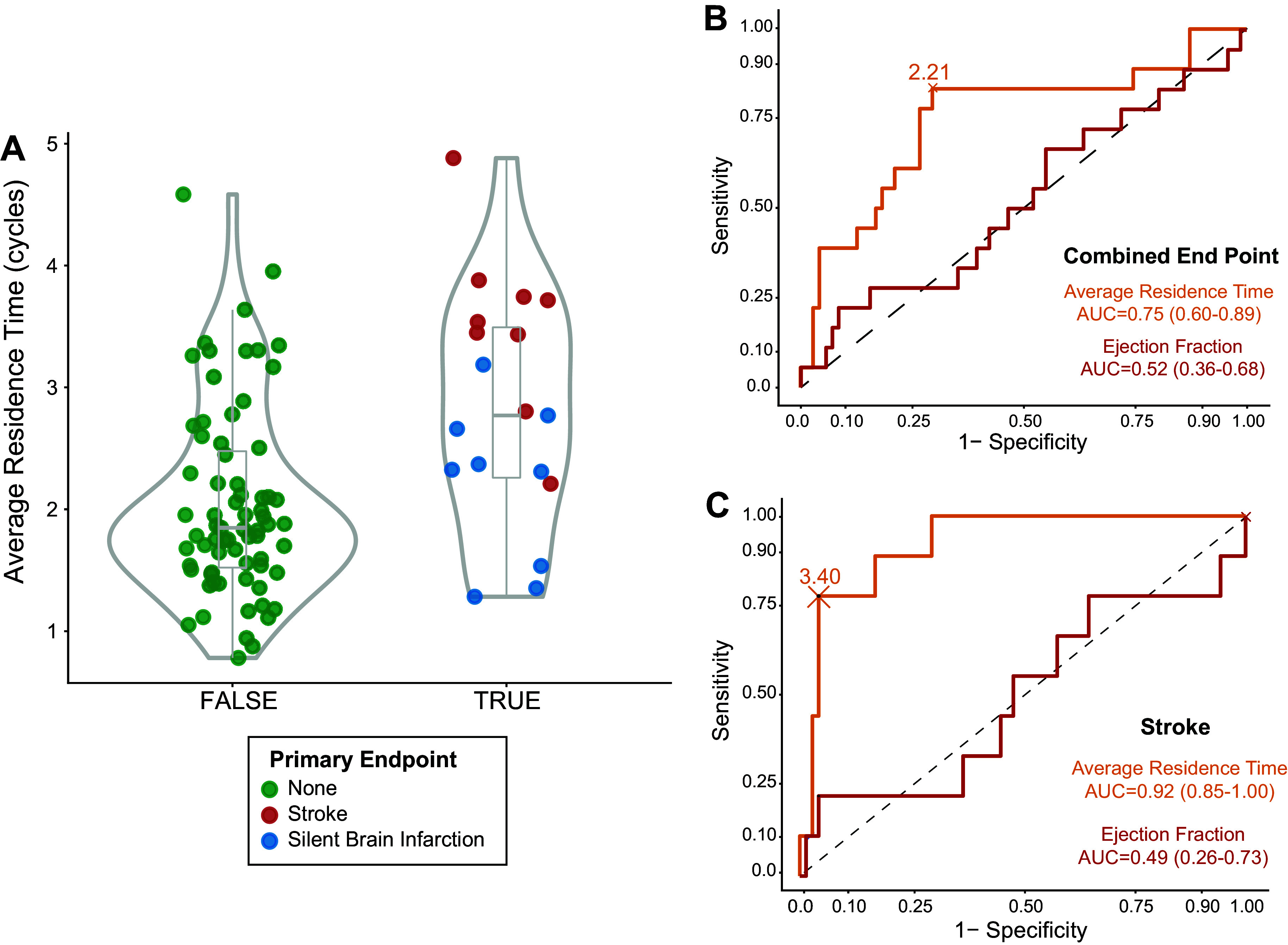
Relationship between average residence time and the primary end point. *A*: violin plot of the distributions of average residence time ($\bar{T_{\text{R}}}$). The primary end point is colored upon its etiology. *B*: receiver-operating characteristic (ROC) curve for the performance of $\bar{T_{\text{R}}}$ for addressing the primary end point. *C*: ROC curve for the performance of $\bar{T_{\text{R}}}\;$ for assessing stroke.

$\bar{T_{\text{R}}}$ performed favorably in identifying the primary end point (AUC = 0.75, 95% CI = 0.61–0.89, OR = 2.83 per cardiac cycle, 95% CI = 1.50–5.34, *P* = 0.001) and remained significantly associated with the primary end point in bivariate models adjusted for covariables such as diabetes, dyslipidemia, hypertension, and creatinine levels (OR in the range of 2.51–2.74, all *P* < 0.01). When accounting only for the relationship with stroke or TIA, the AUC for $\bar{T_{\text{R}}}\;$ was 0.92 (0.85–1.00) with an OR = 7.20, 95% CI = 2.30–22.30, *P* < 0.001 (Fig. 2). Stasis indices for identifying SBIs showed values between the range of 0.58 [0.37–0.79] (for $\bar{T_{\text{R}}}$) and 0.88 [0.72–1.00] (for the stasis timescale of severely stagnant regions).

**Figure 3.**
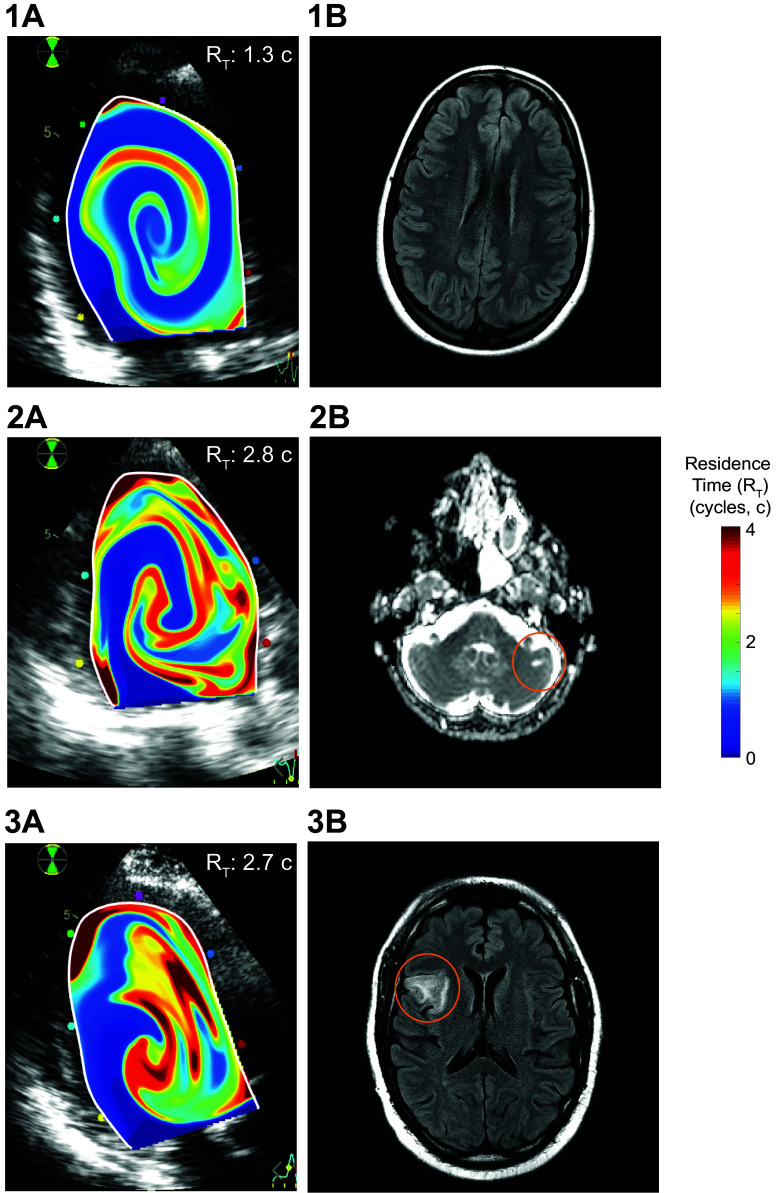
Examples of residence time mapping and brain MR. *1A* and *1B*: residence time (*T*_R_) distribution in the left ventricular (LV) and brain MR of a patient with nonischemic dilated cardiomyopathy (NIDCM) without event. *2A* and *2B* and *3A* and *3B*: *T*_R_ distribution in the LV and brain MR of two patients with NIDCM with a silent brain infarction (SBI, 2*A* and *2B*) and a stroke (3*A* and *3B*). *T*_R_ maps are overlaid on the B-mode echocardiogram.

The overall relationship of the primary end point with $\bar{T_{\text{R}}}\;$ was higher than with other conventional imaging parameters, such as LV EF [AUC = 0.52 (0.36–0.68)], global and apical strain (AUCs of 0.44 and 0.55, respectively, *P* > 0.05 for both), or E-wave penetration index (AUC of 0.62, *P* = 0.07). Local indices of stasis also outperformed conventional metrics: their sizes showed AUCs of 0.70 (0.56–0.84) and 0.73 (0.56–0.89) and their stasis timescales showed AUCs of 0.77 (0.68–0.90) and 0.81 (0.68–0.94), for stagnant regions and severely stagnant regions, respectively (Supplemental Fig. S1). Stasis timescales of both regions were not associated with any conventional index from ultrasound or CMR. The only traditional metric that performed well to assess the predicted primary end point (AUC: 0.71 [0.61–0.86]) and stroke risk (AUC: 0.84 [0.74–0.95]) was the CHA_2_DS_2_-VASc score, although it did not correlate with any of the stasis indices (*R* < 0.12 for all). Figure 3 shows illustrative examples of stasis imaging and cardiac and brain magnetic resonance for a patient with stroke, a patient with SBIs, and a patient free of events.

## DISCUSSION

As far as we know, this is the first study aimed to validate imaging-based biomarkers of intracardiac stasis against the burden of brain infarcts, TIAs, and stroke in a moderately large cohort of patients with nonischemic dilated cardiomyopathy, in the absence of AF. Classic imaging markers of intraventricular thrombosis such as EF were not related to cardioembolic stroke or SBIs in our cohort. Moreover, advanced imaging indices, such as longitudinal strain, which have been proven useful to predict thrombosis in the LA in patients with AF (26), or after STEMI in the LV (13, 27), also showed no relationships with cardioembolic events in our study. In contrast, stasis indices were closely related to ischemic brain events, whether clinical or silent.

### Silent Brain Infarct in NIDCM

In patients with NIDCM, prevalence of SBIs has been disclosed to be as high as 35% (4), and intraventricular thrombosis occurrence has been reported to be around 13%, frequently accompanied by high prevalence of thrombi in the atrium and left atrial appendage. In our study, the prevalence of SBI was 10%, and we did not observe LV mural thrombosis in any patient. This prevalence was lower than previously reported, probably because previous studies included patients with ischemic cardiomyopathy, AF (28), and lower ejection fraction values (29). The fact that we did not identify any case of mural thrombosis by late gadolinium enhancement-CMR, does not exclude a cardioembolism as the etiology of the ischemic events, as the stagnant regions in the cardiac chambers may activate the coagulation cascade without macroscopic thrombosis ever being detected, either because a small mural thrombus is missed or because the thrombotic material embolizes before the patient is scanned.

### Intraventricular Blood Stasis as a Source of Cardioembolism in NIDCM

As one of the three pillars of Virchow’s triad, blood stasis is a recognized key factor for cardiac embolism (30). However, until recently, the assessment of stasis in the heart was qualitatively based only on the visual interpretation of spontaneous contrast from B-mode ultrasound, which is highly dependent on equipment, operator, and factors such as the relative concentrations of red blood cells and fibrinogen (31).

Advances in medical imaging now make it possible to measure time-resolved two- or even three-dimensional flow velocity fields inside the cardiac chambers. The analysis of these datasets using the laws of fluid mechanics allows for quantifying flow-related indices accounting for the transport and stagnation of blood inside the cardiac chambers (21). One of these indices, the residence time, *T*_R_, is particularly well suited to quantify blood stasis, as it can measure the time that fluid particles spend inside a cardiac chamber (10, 32). The performance of *T*_R_ to predict cardioembolism has been recently demonstrated in the setting of STEMI, both in animals, in which an averaged high *T*_R_ and large size of stagnant regions in the LV correlated with the occurrence of high-intensity cerebral signals (12), and in patients, in whom averaged *T*_R_ showed good performance to predict intraventricular thrombus, SBI, and stroke (13). The present study significantly extends these previous findings by confirming that *T*_R_-derived indices may also be a good biomarker of SBI and stroke beyond the specifics found in the infarcted LV.

### Flow Patterns and Stasis in Patients with NIDCM

The diastolic LV swirling flow pattern routes blood entering the chamber to displace the blood volume lingering from previous beats toward the outflow tract (33), helping to minimize the residence time of blood. The changes in chamber size and morphology associated with NIDCM strengthen this pattern, creating larger, more persistent swirling structures as compared with normal hearts (8) (Supplemental Fig. S2 and Supplemental Videos S1 and S2). This mechanism could at least partially compensate for the increase in *T*_R_ concomitant with reduced EF, and differences in flow patterns among patients would explain why EF is not related to adverse events in our cohort. In patients with NIDCM, blood may be trapped inside long-lasting vortices that may endure the entire cardiac cycle. Therefore, accounting for intraventricular stasis in this scenario must consider additional factors beyond the averaged *T*_R_, such as the size and distortion of fluid parcels with long persistence in the LV.

### Limitations

This was a monocentric study, and the number of events was small. Therefore, further validation in larger prospective clinical trials is needed. Alternative etiologies may result in SBIs beyond cardioembolism. To reduce the possibility of secondary sources, we performed comprehensive etiological research in all patients with SBIs and excluded all patients with any history of known or suspected AF. However, small vessel disease or subclinical AF were impossible to address by design and could be responsible for some of identified lesions. Also, the hemodynamic status of each individual patient may have changed between the time of occurrence of the event and imaging. Stasis imaging was based on conventional 2-D transthoracic echocardiography, assuming a planar-flow distribution inside the LV. Although this is an inherent methodological simplification, several studies have validated this hypothesis (34).

### Conclusions

In patients with NIDCM in sinus rhythm, stasis imaging derived from bedside echocardiography is related to the burden of cardioembolic stroke.

## DATA AVAILABILITY

Data supporting this study are available upon request.

## SUPPLEMENTAL DATA

10.6084/m9.figshare.25944277.v2 Supplemental Figs. S1–S2 and Supplemental Videos S1–S2: https://doi.org/10.6084/m9.figshare.25944277.v2.

## GRANTS

This study was supported by the Spanish Society of Cardiology (ISBIDCM), Instituto de Salud Carlos III Grant PACER-1 PI21/00274, and by the European Union via European Regional Development Fund. J.C.d.A. was partially supported by National Heart, Lung, and Blood Institute Grants R01HL158667 and R01HL160024.

## DISCLOSURES

P.M.L., J.C.d.A., and J.B. are inventors of a method for quantifying intracardiac stasis and shear stresses from imaging data under a Patent Cooperation Treaty application (WO2017091746A1). None of the other authors has any conflicts of interest, financial or otherwise, to disclose.

## AUTHOR CONTRIBUTIONS

E.R.G., P.M.L., J.C.d.A., and J.B. conceived and designed research; E.R.G., A.G.M., M.A.E., T.M., J.A.G.d.V., F.D.O., and R. G.d.A. performed experiments; E.R.G., A.F.A., C.P.I., J.C.d.A., J.B., P.M.L., A.G.M., M.A.E., T.M., J.A.G.d.V., F.D.O., and R.G.d.A. analyzed data; P.F.G., A.F.A., C.P.I., J.C.d.A., J.B., P.M.L., A.G.M., M.A.E., T.M., J.A.G.d.V., F.D.O., and R.G.d.A. interpreted results of experiments; E.R.G., J.B., P.M.L., J.A.G.d.V., and M.G.B. prepared figures; E.R.G., P.F.G., C.P.I., J.C.d.A., J.B., P.M.L., M.A.E., and M.G.B. drafted manuscript; P.F.G., A.F.A., C.P.I., J.C.d.A., J.B., P.M.L., and M.G.B. edited and revised manuscript; E.R.G., P.F.G., A.F.A., C.P.I., J.C.d.A., J.B., P.M.L., A.G.M., M.A.E., T.M., J.A.G.d.V., M.G.B., F.D.O., and R.G.d.A. approved the final version of manuscript.
